# Characteristics of Nonthermal Dupree Diffusion on Space-Charge Wave in a Kappa Distribution Plasma Column with Turbulent Diffusion

**DOI:** 10.3390/e22020257

**Published:** 2020-02-24

**Authors:** Myoung-Jae Lee, Young-Dae Jung

**Affiliations:** 1 Department of Physics, Hanyang University, Seoul 04763, Korea; mjlee@hanyang.ac.kr; 2 Research Institute for Natural Sciences, Hanyang University, Seoul 04763, Korea; 3 Department of Applied Physics and Department of Bionanotechnology, Hanyang University, Ansan, Kyunggi-Do 15588, Korea

**Keywords:** nonthermal diffusion, space-charge wave, kappa turbulent plasma

## Abstract

The nonthermal diffusion effects on the dispersion equations of ion-acoustic space-charge wave (SCW) in a nonthermal plasma column composed of nonthermal turbulent electrons and cold ions are investigated based on the analysis of normal modes and the separation of variables. It is found that the real portion of the wave frequency of the SCW in a Maxwellian plasma is greater than that in a nonthermal plasma. It is also found that the magnitude of the damping rate of the SCW decreases with an increase of the spectral index of the nonthermal plasma. It is also shown that the magnitude of the scaled damping rate increases with an increase of the Dupree diffusion coefficient. Moreover, the influence of the nonthermal character of the nonthermal plasma on the damping rate is found to be more significant in turbulent plasmas with higher diffusion coefficient. The variations of the wave frequency and the growth rate due to the characteristics of nonthermal diffusion are also discussed.

## 1. Introduction

In various boundary plasma systems, the propagation and the instability of plasma waves have received considerable attention because they are known to provide useful information on the dispersion characteristics of surface waves as well as the physical characteristics of geometries by examining plasma waves at the interface of bounded plasma systems. Additionally, the dispersion characteristics of surface plasma waves are widely applied in many scientific and technological fields, such as astrophysics, laser physics, materials science, nanoscience, plasma spectroscopy, and space physics [1,2,3,4,5,6,7,8,9,10]. The dispersion relation for surface waves in planar hemispheric and bounded plasma systems can be obtained by adopting the well-known specular reflection condition [9,11,12]. Additionally, the cylindrical beam composition of plasma columns has become of great concern because the space-charge wave (SCW) can support various frequency modes due to the harmonization of the Bessel solution [13]. However, when the dielectric function takes a complex form due to external disturbances, the investigation of the physical properties of surface waves in plasma is quite difficult to solve because of the mathematical complexity of complex analysis. It is known that the motion of plasma electrons will be affected by field variations, because they play an important role in the binary collision in plasma and external disturbances caused by plasma turbulence [14,15,16]. In addition, nonthermal plasmas are frequently found in many plasma environments because the external radiation field is combined with the plasma system [17]. The generalized kappa distribution for the plasma that possesses a non-Maxwellian tail is often used as a powerful tool for investigating nonthermal effects on the collision and radiation processes in the nonthermal plasmas [18,19,20]. As far as we know, the diffusion effect on the stability of SCWs of nonthermal plasma columns with the kappa distribution and turbulent diffusion has not been reported yet. This study was conducted to investigate the theoretical aspects of the nonthermal plasma. Therefore, the methods of normal mode analysis and separation of variables are adopted in this paper to examine the geometrical effect on ion acoustic SCWs in a nonthermal plasma with the kappa distribution and turbulent diffusion due to variation in charge density. Then, from the perspective of the spectral index and the Dupree diffusion parameters of the kappa distribution, the dispersion relation for the SCW is obtained. The variations of the wave frequency and the growth rate due to the nonthermal diffusion are also discussed.

## 2. Theory and Calculations

The kappa (or Lorentzian) distribution function in nonthermal plasmas is expressed by the power-law velocity form [17,18,19,20] as follows:

(1) $$f_{L}(v_{j})=\frac{\Gamma (\kappa +1)}{\Gamma (\kappa -1/2)}{\left[\frac{m_{j}}{2\pi \kappa E_{Lj}\left(\kappa \right)}\right]}^{3/2}{\left[1+\frac{m_{j}v_{j}^{2}}{2\kappa E_{Lj}\left(\kappa \right)}\right]}^{-(\kappa +1)},$$

where $v_{j}$ is the velocity of the plasma; κ is the spectral index (κ>3/2) of the kappa distribution; Γ(κ) is the Gamma function; $m_{j}$ is the mass of plasma; $E_{Lj}(\kappa )=\beta^{2}(\kappa )E_{Tj}$ is the characteristic energy with $\beta (\kappa )={\left[(2\kappa -3)/2\kappa \right]}^{1/2}$ and $E_{Tj}=k_{B}T_{j}$, where $k_{B}$ is the Boltzmann constant; and $T_{j}$ is the plasma temperature. The subscript *j* denotes the species *j* ( = *e*, *i* for electron and ions, respectively) in the kappa distribution. If κ→∞, i.e., in the absence of the external radiation field, the kappa distribution function $f_{L}$ becomes the Maxwellian distribution $f_{M}$ such as $f_{L,\kappa \to \infty }(v_{j})=f_{M}(v_{j})\propto \exp(-mv_{j}^{2}/2E_{Tj})$ [17]. In addition, the effective screening length $\lambda_{Lj}(\kappa )$ [20] of the species *j* in the nonthermal plasma is expressed by $\lambda_{Lj}(\kappa )=\alpha (\kappa )\lambda_{Dj}$, where $\alpha (\kappa )={\left[(2\kappa -3)/(2\kappa -1)\right]}^{1/2}$ is the kappa parameter [18] and $\lambda_{Dj}(=\sqrt{k_{B}T_{j}/4\pi n_{j}q_{j}^{2}})$ is the standard Debye length of the species *j* in Maxwellian plasmas, with $n_{j}$ and $q_{j}$ being the density and the charge of the species *j*. The kappa parameter α(κ) represents the measure of the fraction of nonthermal population in the kappa tail. Based on the Shukla–Spatschek turbulence (SST) model [15], including the nonthermal shielding effect and the Dupree far-field term $\left[r>\lambda_{Le}(\kappa )\right]$ with the condition of $v<v_{Le}(\kappa )$ due to the fluctuation of the external field, the electron–electron interaction potential $V_{L}^{SST}(r)$ is found to be

(2) $$V_{L}^{SST}(r)=\frac{e^{2}}{r}\exp\left[-\frac{r}{\lambda_{Le}(\kappa )}\right]+\frac{\sqrt{8}e^{2}}{\sqrt{\pi }r}\cos\theta {\left[\frac{\lambda_{Le}(\kappa )}{r}\right]}^{2}\left[\frac{v}{v_{Le}(\kappa )}\right]\left[1-\frac{4\sqrt{\pi }}{9}\frac{D}{v_{Le}^{4}(\kappa )}r\right],$$

where *r* is the distance between the projectile electron and the target ion, θ is the angle between **r** and **v**, $v_{L}$ is the characteristic velocity in the kappa distribution, *D* is the Dupree diffusion coefficient [15] owing to the Gaussian-Spatial-Diffusion (GSD) type correction term $F_{GSD}(k,t)\propto e^{-k^{2}Dt^{3}/3}$ on account of the random walk caused by the plasma turbulence, *k* is the wave number, and *t* is the time. Hence, it is expected that the dispersions $\langle {\left(\Delta z\right)}^{2}\rangle$ in the spatial position increase as the cube of the elapsed time such as $\langle {\left(\Delta z\right)}^{2}\rangle =2Dt^{3}/3$ [21]. It should be noted that Gaussian diffusion due to turbulent field fluctuations is quite similar to the spread by short-range collisions because Coulomb collisions produce random walkways in the speed space [21]. Since we are interested in investigating the nonthermal effects on the ion acoustic SCW in Lorentz turbulence (LT) plasma column, which consists of nonthermal electrons and cold ions, we consider the plasma dielectric function $\varepsilon_{LT}(\omega ,k)$ in the frequency range where the phase velocity far exceeds the characteristic velocity of ion but is much less than that of the electron, i.e., $v_{Li}<<\omega /k<<v_{Le}$ where ω is the wave frequency, and $v_{Lj}$ is the characteristic velocity of the species *j*

(3) $$\varepsilon_{LT}(\kappa ,\omega ,k)=1+\frac{1}{k^{2}\lambda_{Le}^{2}(\kappa )}-\frac{\omega_{pi}^{2}}{\omega^{2}}+i\left[\sqrt{\frac{\pi }{2}}+\frac{4D}{27kv_{Le}^{3}(\kappa )}\right]\frac{\omega }{k^{3}\lambda_{Le}^{2}(\kappa )v_{Le}(\kappa )},$$

where $\omega_{pi}={\left(4\pi n_{i}q_{i}^{2}/m_{i}\right)}^{1/2}$ is the plasma frequency of the ion. In order to specify the level of turbulence, it should be noted that the energy density of the fluctuating electric field is smaller than the characteristic energy $E_{Lj}(\kappa )$ in the kappa distribution. In this work, we neglect the influence of wave absorption or emission on the broadening since we are interested in the frequency range where the phase velocity far exceeds the characteristic velocity of ion but is much less than that of the electron. We now consider a nonthermal plasma consisting of electrons and ions in a cylindrically bounded plasma column with turbulent diffusion along the *z*-axis of a cylindrical coordinate system (ρ,θ,z). Here, we assume that the nonthermal plasma is azimuthally symmetric, such as in ∂/∂θ=0; there is no external magnetic field; and the curl of electric field is null, i.e., the perturbation is electrostatic. Under this circumstance, the motion of plasmas in the planar (ρ,θ) plane can be ignored and the *z*-directional propagation of waves will be important. In an unmagnetized plasma, the equations of continuity and momentum for the species *j* of plasma particle are given by

(4) $$\frac{\partial n_{j}}{\partial t}+\nabla \cdot (n_{j}v_{j})=0,$$

(5) $$n_{j}\left(\frac{\partial v_{j}}{\partial t}+v_{j}\cdot \nabla v_{j}\right)=-\frac{1}{m_{j}}\nabla P_{j}-\frac{q_{j}n_{j}}{m_{j}}\nabla \varphi ,$$

with Poisson’s equation $\nabla^{2}\varphi =-4\pi \sum_{j=e,i}q_{j}n_{j}$, where $n_{j}$, $v_{j}$, $P_{j}$, $q_{j}$, and φ are the density, velocity, pressure, and electrostatic potential, respectively. To obtain the dispersion relation for the SCW, a small perturbation will be expressed as follows: $n_{j}=n_{0j}+n_{1j}$, $v_{j}=v_{0j}+v_{1j}$, and $\varphi =\varphi_{1}$, where the subscript 0 denotes the equilibrium quantity and the subscript 1 for the small perturbation from its equilibrium values of the given quantities. In a cylindrical coordinate system, the perturbed density, velocity, and electrostatic potential can be written as wave-like quantities [22,23], such as

(6) $$\left(\begin{array}{c} {\bar{n}}_{1j}(r,t)\\ v_{1j}(r,t)\\ \varphi_{1}(r,t) \end{array}\right)=\left(\begin{array}{c} {\tilde{n}}_{1j}(\rho )\\ {\tilde{v}}_{1j}(\rho )\\ {\tilde{\varphi }}_{1}(\rho ) \end{array}\right)\times \exp\left[i(\xi \theta +k_{∥}z-\omega t)\right],$$

where ${\tilde{n}}_{1j}(\rho )$, ${\tilde{v}}_{1j}(\rho )$, and ${\tilde{\varphi }}_{1}(\rho )$ are the perturbation quantities in the transverse planar plane; ξ is the azimuthal wave number, $k_{∥}[={\left(k^{2}-k_{⊥}^{2}\right)}^{1/2}]$ is the propagation wave number along the axial *z*-direction of the cylindrical plasma column; and $k_{⊥}$ is the transverse wave number. For $\varphi_{1}$ to be uniform, it is necessary that the parameter ξ must be an integer [24]. From Equations (4)–(6) with Poisson’s equation and Equation (3) for the plasma dielectric function, the differential equation for the perturbed transverse electrostatic potential ${\tilde{\varphi }}_{1}(\rho )$ can be represented by the following form:

(7) $$\frac{d^{2}{\tilde{\varphi }}_{1}(\rho )}{d\rho^{2}}+\frac{1}{\rho }\frac{d{\tilde{\varphi }}_{1}(\rho )}{d\rho }+\left[\mu^{2}(\kappa ,\omega ,k_{∥})-\frac{\xi^{2}}{\rho^{2}}\right]{\tilde{\varphi }}_{1}(\rho )=0,$$

where the separation parameter $\mu^{2}(\kappa ,\omega ,k_{∥})[=-k_{∥}^{2}\varepsilon_{LT}(\kappa ,\omega ,k_{∥})]$ is represented by

(8) $$\mu^{2}(\kappa ,\omega ,k_{∥})=-k_{∥}^{2}\left\{1+\frac{1}{k_{∥}^{2}\lambda_{Le}^{2}(\kappa )}-\frac{\omega_{pi}^{2}}{\omega^{2}}+i\left[\sqrt{\frac{\pi }{2}}+\frac{4D}{27k_{∥}v_{Le}^{3}(\kappa )}\right]\frac{\omega }{k_{∥}^{3}\lambda_{Le}^{2}(\kappa )v_{Le}(\kappa )}\right\}.$$

The general solution of Equation (6) is then obtained by ${\tilde{\varphi }}_{1}(\rho )=A_{\xi }J_{\xi }(\zeta \rho )+B_{\xi }N_{\xi }(\zeta \rho )$, where $A_{\xi }$ and $B_{\xi }$ are constants to be determined by the boundary conditions, $J_{\xi }(\zeta \rho )$ is the ξth-order Bessel function of the first kind, and $N_{\xi }(\zeta \rho )$ is the ξth-order Neumann function. However, we have the ξ=0 perturbation [25] for the azimuthally symmetric cylindrical system since $(\partial^{2}\varphi_{1}/\partial \theta^{2})/\varphi_{1}=-\xi^{2}$. Hence, the general solution for the azimuthally symmetric plasma column is given by ${\tilde{\varphi }}_{1}(\rho )=A_{0}J_{0}(\zeta \rho )+B_{0}N_{0}(\zeta \rho )$ since we set ξ=0. At the origin, i.e.,ρ=0, we should have the regular solutions and the perturbed electrostatic potential ${\tilde{\varphi }}_{1}(0)$ should be finite. Therefore, we set $B_{0}=0$. Additionally, the perturbed electrostatic potential ${\tilde{\varphi }}_{1}(\rho )$ must be zero on the surface of the cylindrical plasma column at ρ=R, i.e., $J_{0}(\zeta R)=0$. Hence, the parameter ζ is determined by $\zeta =\alpha_{0n}/R$, where $\alpha_{0n}$ is the *n*th zero of the Bessel function of order zero ($\alpha_{01}=2.4048$, $\alpha_{02}=5.5201$, $\alpha_{03}=8.6537$, …), i.e., $J_{0}(\alpha_{0n})=0$. The dispersion relation $\hat{D}(\kappa ,\omega ,k_{∥})$ is then written as

(9) $$\begin{array}{ll} \hat{D}(\kappa ,\omega ,k_{∥}) & ={\hat{D}}_{R}(\kappa ,\omega ,k_{∥})+i{\hat{D}}_{I}(\kappa ,\omega ,k_{∥})\\ \; & =1+\frac{1}{k_{∥}^{2}\lambda_{Le}^{2}(\kappa )}+\frac{\alpha_{0n}^{2}}{k_{∥}^{2}R^{2}}-\frac{\omega_{pi}^{2}}{\omega^{2}}+i\left[\sqrt{\frac{\pi }{2}}+\frac{4D}{27k_{∥}v_{Le}^{3}(\kappa )}\right]\frac{\omega }{k_{∥}^{3}\lambda_{Le}^{2}(\kappa )v_{Le}(\kappa )}=0, \end{array}$$

where ${\hat{D}}_{R}(\omega ,k_{∥})$ and ${\hat{D}}_{I}(\omega ,k_{∥})$ are the real and the imaginary parts of the dispersion function $\hat{D}(\omega ,k_{∥})$, respectively. By letting $\omega (k_{∥})=\omega_{R}(k_{∥})+i\gamma (k_{∥})$ for real $k_{∥}$ in the dispersion function $\hat{D}(\omega ,k_{∥})$, i.e., $\hat{D}(\omega_{R}+i\gamma ,k_{∥})=0$, and assuming $\left|\gamma \right|<<\omega_{R}$ with the Taylor expansion, the real part ${\bar{\omega }}_{R}(=\omega_{R}/\omega_{pi})$ of the scaled frequency of the SCW in a nonthermal plasma column with turbulent diffusion due to the fluctuation of the charge density is found to be

(10) $${\bar{\omega }}_{R}(\kappa ,{\bar{k}}_{∥},\bar{R})=\frac{1}{\sqrt{1+\frac{2\kappa -1}{2\kappa -3}\frac{1}{{\bar{k}}_{∥}^{2}}\left(1+\frac{2\kappa -3}{2\kappa -1}\frac{\alpha_{0n}^{2}}{{\bar{R}}^{2}}\right)}},$$

since ${\hat{D}}_{R}(\kappa ,\omega_{R},k_{∥})=0$, where $\omega_{R}$ and γ are, respectively, the real and the imaginary parts of the frequency of the SCW; ${\bar{k}}_{∥}(\equiv k_{∥}\lambda_{De})$ is the scaled axial wave number; and $\bar{R}(\equiv R/\lambda_{De})$ is the scaled radius of the cylindrical plasma column. The detailed derivations of the scaled frequency and the scaled damping rate are given in Appendix A. For large wave numbers, ${\bar{k}}_{∥}>>1$, the real part $\omega_{R}$ of the frequency saturates to the standing wave mode with the ion plasma frequency $\omega_{pi}$ since Equation (9) is the ion-acoustic SCW in a nonthermal plasma column with turbulent diffusion. In a Maxwellian plasma column, i.e., κ→∞, the real part of the frequency becomes ${\bar{\omega }}_{R}({\bar{k}}_{∥})\to {\bar{k}}_{∥}/{\left(1+{\bar{k}}_{∥}^{2}+\alpha_{0n}^{2}/{\bar{R}}^{2}\right)}^{1/2}$ [26]. If we set ξ≠0 in Equation (6), the solution is related to $J_{\xi }(\zeta \rho )$ so that the real part of frequency can be written in terms of the ratio $J_{\xi }(\zeta R)/{J^{\prime }}_{\xi }(\zeta R)$ [27]. Hence, the dipole resonance modes can exist for ξ=1 case. The detailed discussions on the Bessel functions are given in Appendix B. From Equation (9) with $\omega =\omega_{R}+i\gamma$, the damping rate γ for the small perturbation by using the Taylor expansion is given by $\gamma =-{\overset{⌢}{D}}_{I}(\kappa ,\omega_{R},k_{∥})/[\partial {\overset{⌢}{D}}_{R}(\kappa ,\omega_{R},k_{∥})/\partial \omega_{R}]$. Hence, the scaled damping rate $\bar{\gamma }(=\gamma /\omega_{pi})$ of the SCW in a nonthermal plasma column with turbulent diffusion can be obtained by a perturbational algebra such as

(11) $$\bar{\gamma }(\kappa ,\bar{D},{\bar{k}}_{∥},\bar{R})=-\frac{\sqrt{\frac{\pi }{8}}+\frac{2}{27}{\left(\frac{2\kappa }{2\kappa -3}\right)}^{3}\frac{\bar{D}}{{\bar{k}}_{∥}}}{{\bar{\omega }}_{pe}{\bar{k}}_{∥}^{3}{\left(\frac{2\kappa -3}{2\kappa -1}\right)}^{3}\left[1+\frac{2\kappa -1}{2\kappa -3}\frac{1}{{\bar{k}}_{∥}^{2}}\left(1+\frac{2\kappa -3}{2\kappa -1}\frac{\alpha_{0n}^{2}}{{\bar{R}}^{2}}\right)\right]},$$

where ${\bar{\omega }}_{pe}\equiv \omega_{pe}/\omega_{pi}$ and $\bar{D}(\equiv D\lambda_{De}/v_{Te}^{3})$ is the scaled Dupree diffusion coefficient. Very recently, the nonthermal turbulence and the plasma screening effects were investigated in the electron–ion collision process in kappa turbulent plasmas [28]. However, the influence of the nonthermal turbulence on the stability of the SCW in a nonthermal plasma with turbulent diffusion has not been investigated yet. Hence, it is expected that Equations (9) and (10) would provide useful information on the propagation of the SCW in nonthermal turbulent plasmas. Recently, a new form of the nonthermal distribution function known as the (*r*, *q*) distribution, which consists of two spectral indices, has been used to explore the wave properties in non-Maxwellian plasmas [29]. In addition, since dust grains are ubiquitous in numerous astrophysical and laboratory plasmas and dust grains, the physical characteristics of strongly coupled plasmas composed of electrons, ions, and charged dust grains have been widely used in nano-science and technology [30,31,32,33,34]. Hence, the investigation of dispersion properties of ion-acoustic SCWs in the strongly coupled dusty and plasma column with (*r*, *q*) distribution and turbulent diffusion will be treated elsewhere.

## 3. Discussions

**Figure 1:**
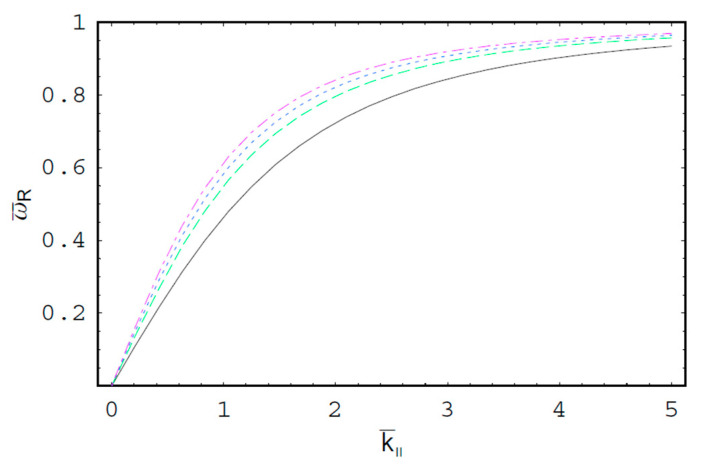
The scaled real part ${\bar{\omega }}_{R}$ of the wave frequency of the space-charge wave (SCW) as a function of the scaled axial wave number ${\bar{k}}_{∥}$ for $\bar{R}=3$ and the first-harmonic, i.e., $\alpha_{01}=2.4048$. The solid line is the case of κ=2. The dashed line is the case of κ=3. The dotted line is the case of κ=5. The dash-dot is the case of κ→∞, i.e., Maxwellian case.

**Figure 2:**
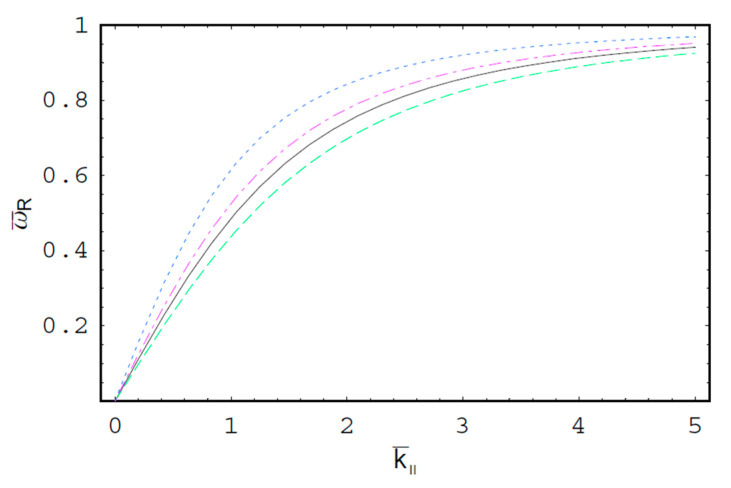
The scaled real part ${\bar{\omega }}_{R}$ of the wave frequency of the SCW as a function of the scaled axial wave number ${\bar{k}}_{∥}$ for $\bar{R}=5$. The solid line is the case of κ=2 and the first-harmonic, i.e., $\alpha_{01}=2.4048$. The dashed line is the case of κ=2 and the second-harmonic, i.e., $\alpha_{02}=5.5201$. The dotted line is the case of κ=4 and the first-harmonic, i.e., $\alpha_{01}=2.4048$. The dash-dot is the case of κ=4 and the second-harmonic, i.e., $\alpha_{02}=5.5201$.

**Figure 3:**
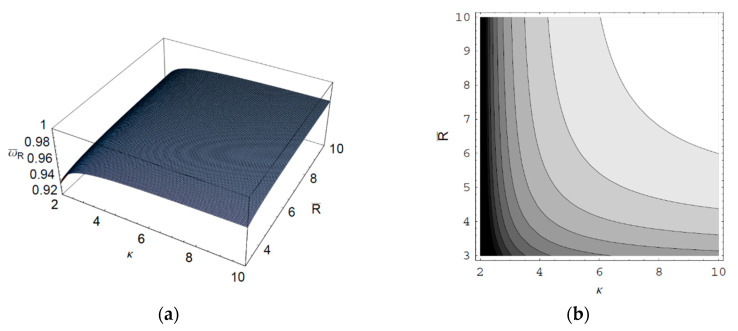
(**a**) Surface plot; (**b**) Contour plot of the scaled real part ${\bar{\omega }}_{R}$ of the wave frequency of the SCW as a function of the spectral index κ and the scaled radius $\bar{R}$ of the cylindrical plasma column for ${\bar{k}}_{∥}=5$ and the first-harmonic, i.e., $\alpha_{01}=2.4048$.

**Figure 4:**
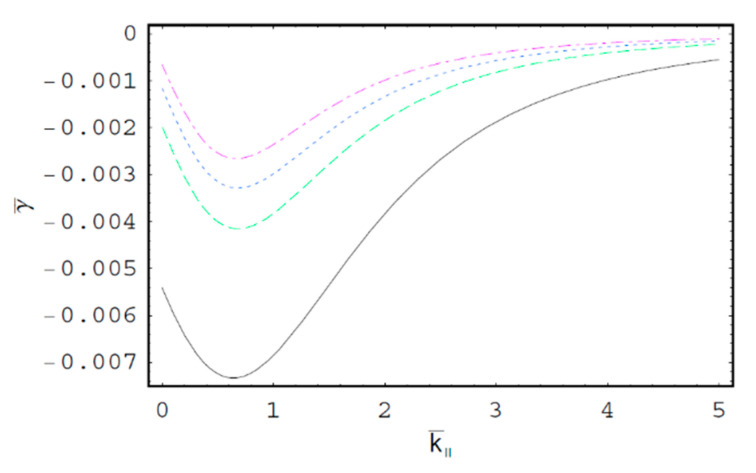
The scaled imaginary part of the wave frequency, i.e., the scaled damping rate $\bar{\gamma }$, as a function of the scaled axial wave number ${\bar{k}}_{∥}$ for $\bar{D}=1$; $\bar{R}=3$; and the first-harmonic, i.e., $\alpha_{01}=2.4048$. The solid line is the case of κ=2. The dashed line is the case of κ=3. The dotted line is the case of κ=5. The dash-dot is the case of κ→∞, i.e., Maxwellian case.

**Figure 5:**
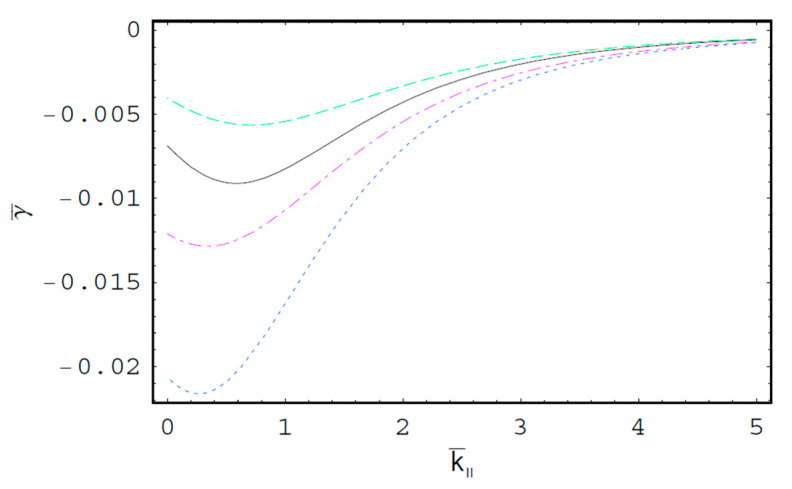
The scaled damping rate $\bar{\gamma }$ of the SCW as a function of the scaled axial wave number ${\bar{k}}_{∥}$ for κ=2 and $\bar{R}=5$. The solid line is the case of $\bar{D}=1$ and the first-harmonic, i.e., $\alpha_{01}=2.4048$. The dashed line is the case of $\bar{D}=1$ and the second-harmonic, i.e., $\alpha_{02}=5.5201$. The dotted line is the case of $\bar{D}=3$ and the first-harmonic, i.e., $\alpha_{01}=2.4048$. The dash-dot is the case of $\bar{D}=3$ and the second-harmonic, i.e., $\alpha_{02}=5.5201$.

**Figure 6:**
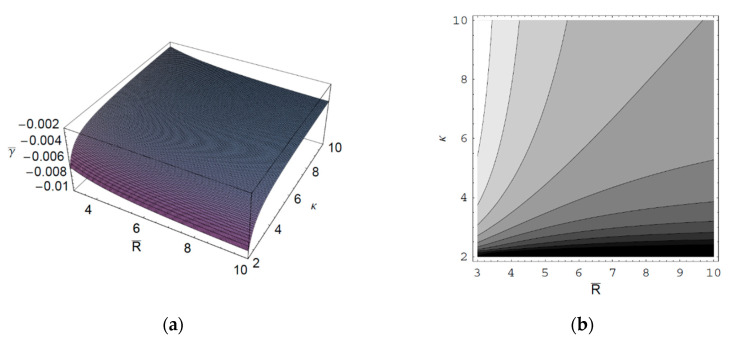
(**a**) Surface plot; (**b**) Contour plot of the scaled damping rate $\bar{\gamma }$ of the SCW as a function of the scaled radius $\bar{R}$ of the cylindrical plasma column and the spectral index κ for ${\bar{k}}_{∥}=1$ and $\bar{D}=1$.

**Figure 7:**
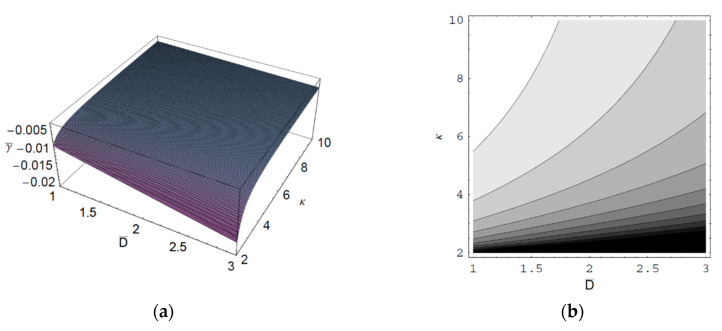
(**a**) Surface plot; (**b**) Contour plot of the scaled damping rate $\bar{\gamma }$ of the SCW as a function of the scaled diffusion coefficient $\bar{D}$ and the spectral index κ for ${\bar{k}}_{∥}=1$ and $\bar{R}=5$.

Figure 1 shows the real part of the scaled wave frequency of the SCW, ${\bar{\omega }}_{R}$, as a function of the scaled axial wave number ${\bar{k}}_{∥}$ for various values of the spectral index κ. It is shown that ${\bar{\omega }}_{R}$ saturates as ${\bar{k}}_{∥}$ increases. We also see that ${\bar{\omega }}_{R}$ increases with an increase of the spectral index κ. Hence, it is found that ${\bar{\omega }}_{R}$ in a thermal plasma is greater than that in a nonthermal plasma since the nonthermal character of the nonthermal plasma suppresses the wave frequency of the SCW. It is also found that the nonthermal effect on ${\bar{\omega }}_{R}$ is more significant in intermediate domain of ${\bar{k}}_{∥}$. Figure 2 shows ${\bar{\omega }}_{R}$ as a function of ${\bar{k}}_{∥}$ for various values of the harmonic mode $\alpha_{0n}$. As we can see in this figure, ${\bar{\omega }}_{R}$ decreases with an increase of the order-*n* of the harmonic mode $\alpha_{0n}$. It is also shown that the influence of the harmonic mode $\alpha_{0n}$ on ${\bar{\omega }}_{R}$ is significant in the intermediate domain of ${\bar{k}}_{∥}$. Figure 3 represents the surface plot of ${\bar{\omega }}_{R}$ as a function of the spectral index κ and the scaled radius $\bar{R}$ of the cylindrical plasma column. As can be seen, ${\bar{\omega }}_{R}$ increases with an increase of $\bar{R}$ of the cylindrical plasma column. In addition, it is found that the geometric effect on ${\bar{\omega }}_{R}$ increases with an increase of κ. Figure 4 shows the scaled imaginary part of the wave frequency, i.e., the scaled damping rate $\bar{\gamma }$, as a function of ${\bar{k}}_{∥}$ for various values of κ. As we see, the magnitude of $\bar{\gamma }$ decreases with an increase of κ. It is also shown that the nonthermal effect on $\bar{\gamma }$ is significant in the minimum region of ${\bar{k}}_{∥}$. Hence, we have found that the scaled damping rate in a thermal plasma has the smallest value of the damping rate of the SCW. Figure 5 represents $\bar{\gamma }$ as a function of ${\bar{k}}_{∥}$ for various values of the harmonic mode $\alpha_{0n}$ and the scaled diffusion coefficient $\bar{D}$. As shown in this figure, the magnitude of $\bar{\gamma }$ decreases with an increase of $\alpha_{0n}$. In addition, it is shown that the magnitude of $\bar{\gamma }$ increases with an increase of $\bar{D}$. Hence, it is found that the influence of the diffusion strongly suppresses the SCW due to the diffusion damping. It is interesting to note that the minimum position of $\bar{\gamma }$ is shifted to the higher wave number region with an increase of $\bar{D}$. Moreover, the influence of the diffusion on $\bar{\gamma }$ decreases with an increase of ${\bar{k}}_{∥}$. Figure 6 represents surface plot of $\bar{\gamma }$ as a function of the scaled radius $\bar{R}$ and the spectral index κ. As can be seen, the magnitude of $\bar{\gamma }$ increases with an increase of $\bar{R}$ of the cylindrical plasma column. Figure 7 represents surface plot of $\bar{\gamma }$ as a function of $\bar{D}$ and κ. As can be seen, the magnitude of $\bar{\gamma }$ increases with an increase of $\bar{D}$. It is also shown that the influence of the nonthermal character of the nonthermal plasma on $\bar{\gamma }$ is more significant in turbulent plasmas with higher diffusion coefficients.

## 4. Summary

In this work, we investigated the nonthermal diffusion effects on the dispersion properties of ion acoustic SCWs in a nonthermal plasma column with turbulent diffusion, which contains nonthermal electrons and cold ions by employing the normal mode analysis and the method of separation of variables. We have found that the real part of the wave frequency of the SCW in a Maxwellian plasma is greater than that in a nonthermal plasma. We have also found that the magnitude of the damping rate of the SCW decreases with an increase of the spectral index. The magnitude of the scaled damping rate is increased with an increase of the Dupree coefficient, since the magnitude of the scaled damping rate is given by $\left|\bar{\gamma }(\bar{D}=0)\right|=\sqrt{\pi /8}{\left({\bar{\omega }}_{pe}{\bar{k}}_{∥}^{3}\right)}^{-1}{\left(\frac{2\kappa -3}{2\kappa -1}\right)}^{-3}{\left[1+\frac{2\kappa -1}{2\kappa -3}\frac{1}{{\bar{k}}_{∥}^{2}}\left(1+\frac{2\kappa -3}{2\kappa -1}\frac{\alpha_{0n}^{2}}{{\bar{R}}^{2}}\right)\right]}^{-1}$ when the Dupree coefficient is zero. Furthermore, we have found that the effect of the nonthermal character on the damping rate for the SCW in the nonthermal plasma is more significant in turbulent plasma as the Dupree diffusion coefficient increases. Therefore, it is expected that surface waves in a nonthermal plasma would be more difficult to propagate than surface waves in a thermal plasma. Hence, if we measure the wave propagation distance, we can extract the information on the degree of nonthermal character of the plasma. It is also shown that the magnitude of the scaled damping rate increases with an increase of the Dupree diffusion coefficient. From this work, we have found that the influence of nonthermal Dupree diffusion plays a significant role in in a nonthermal plasma with turbulent diffusion column. These results would provide useful information on the stability and the nonthermal effect of SCWs in a kappa turbulent plasma.

## Appendix A. Dispersion Relation

The dispersion relation $\hat{D}(\kappa ,\omega ,k_{∥})\left[={\hat{D}}_{R}(\kappa ,\omega ,k_{∥})+i{\hat{D}}_{I}(\kappa ,\omega ,k_{∥})\right]$ is given by

(A1) $$\hat{D}(\kappa ,\omega ,k_{∥})=1+\frac{1}{k_{∥}^{2}\lambda_{Le}^{2}(\kappa )}+\frac{\alpha_{0n}^{2}}{k_{∥}^{2}R^{2}}-\frac{\omega_{pi}^{2}}{\omega^{2}}+i\left[\sqrt{\frac{\pi }{2}}+\frac{4D}{27k_{∥}v_{Le}^{3}(\kappa )}\right]\frac{\omega }{k_{∥}^{3}\lambda_{Le}^{2}(\kappa )v_{Le}(\kappa )}=0,$$

where ${\hat{D}}_{R}(\kappa ,\omega ,k_{∥})$ is the real part of the dispersion function

(A2) $${\hat{D}}_{R}(\kappa ,\omega ,k_{∥})=1+\frac{1}{k_{∥}^{2}\lambda_{Le}^{2}(\kappa )}+\frac{\alpha_{0n}^{2}}{k_{∥}^{2}R^{2}}-\frac{\omega_{pi}^{2}}{\omega^{2}},$$

and ${\hat{D}}_{I}(\kappa ,\omega ,k_{∥})$ is the imaginary part of the dispersion function

(A3) $${\hat{D}}_{I}(\kappa ,\omega ,k_{∥})=\left[\sqrt{\frac{\pi }{2}}+\frac{4D}{27k_{∥}v_{Le}^{3}(\kappa )}\right]\frac{\omega }{k_{∥}^{3}\lambda_{Le}^{2}(\kappa )v_{Le}(\kappa )}.$$

By letting $\omega (k_{∥})=\omega_{R}(k_{∥})+i\gamma (k_{∥})$ for real $k_{∥}$ in the dispersion function $\hat{D}(\omega ,k_{∥})$, i.e., $\hat{D}(\omega_{R}+i\gamma ,k_{∥})=0$ and assuming $\left|\gamma \right|<<\omega_{R}$ with the Taylor expansion, we have

(A4) $$\hat{D}(\kappa ,\omega_{R}+i\gamma ,k_{∥})\approx {\hat{D}}_{R}(\kappa ,\omega_{R},k_{∥})+i\gamma \frac{\partial {\hat{D}}_{R}(\kappa ,\omega_{R},k_{∥})}{\partial \omega_{R}}+i{\hat{D}}_{I}(\kappa ,\omega_{R},k_{∥})+\cdots =0.$$

From Equations (A2) and (A4), the real part of the dispersion relation is found to be

(A5) $${\hat{D}}_{R}(\kappa ,\omega_{R},k_{∥})=1+\frac{1}{k_{∥}^{2}\lambda_{Le}^{2}(\kappa )}+\frac{\alpha_{0n}^{2}}{k_{∥}^{2}R^{2}}-\frac{\omega_{pi}^{2}}{\omega_{R}^{2}}=0.$$

From Equation (A5), the real part ${\bar{\omega }}_{R}(=\omega_{R}/\omega_{pi})$ of the scaled frequency of the SCW in a nonthermal plasma column with turbulent diffusion due to the fluctuation of the charge density is obtained by

(A6) $${\bar{\omega }}_{R}(\kappa ,{\bar{k}}_{∥},\bar{R})=\frac{1}{\sqrt{1+\frac{2\kappa -1}{2\kappa -3}\frac{1}{{\bar{k}}_{∥}^{2}}\left(1+\frac{2\kappa -3}{2\kappa -1}\frac{\alpha_{0n}^{2}}{{\bar{R}}^{2}}\right)}},$$

where ${\bar{k}}_{∥}(\equiv k_{∥}\lambda_{De})$ is the scaled axial wave number, and $\bar{R}(\equiv R/\lambda_{De})$ is the scaled radius of the cylindrical plasma column. From Equation (A4), the damping rate γ for the small perturbation can be written as

(A7) $$\gamma (\kappa ,\omega_{R},k_{∥})=-\frac{{\overset{⌢}{D}}_{I}(\kappa ,\omega_{R},k_{∥})}{\partial {\overset{⌢}{D}}_{R}(\kappa ,\omega_{R},k_{∥})/\partial \omega_{R}}.$$

From Equations (A3), (A5), (A6), and (A7), the scaled damping rate $\bar{\gamma }(=\gamma /\omega_{pi})$ of the SCW in a nonthermal plasma with turbulent diffusion column is found to be

(A8) $$\bar{\gamma }(\kappa ,\bar{D},{\bar{k}}_{∥},\bar{R})=-\frac{\sqrt{\frac{\pi }{8}}+\frac{2}{27}{\left(\frac{2\kappa }{2\kappa -3}\right)}^{3}\frac{\bar{D}}{{\bar{k}}_{∥}}}{{\bar{\omega }}_{pe}{\bar{k}}_{∥}^{3}{\left(\frac{2\kappa -3}{2\kappa -1}\right)}^{3}\left[1+\frac{2\kappa -1}{2\kappa -3}\frac{1}{{\bar{k}}_{∥}^{2}}\left(1+\frac{2\kappa -3}{2\kappa -1}\frac{\alpha_{0n}^{2}}{{\bar{R}}^{2}}\right)\right]},$$

where ${\bar{\omega }}_{pe}\equiv \omega_{pe}/\omega_{pi}$ and $\bar{D}\equiv D\lambda_{De}/v_{Te}^{3}$.

## Appendix B. Bessel Functions

Bessel’s differential equation [35] is given by

(A9) $$x^{2}\frac{d^{2}y(x)}{dx^{2}}+x\frac{dy(x)}{dx}+\left(x^{2}-\xi^{2}\right)y(x)=0.$$

The solution to Equation (A9) is represented by $y(x)=A_{\xi }J_{\xi }(x)+B_{\xi }N_{\xi }(x)$, where $A_{\xi }$ and $B_{\xi }$ are constants to be determined by the boundary conditions, $J_{\xi }(x)$ is the ξth-order Bessel function of the first kind [35]

(A10) $$J_{\xi }(x)=\sum_{m=0}^{\infty }\frac{{\left(-1\right)}^{m}}{m!\Gamma (m+\xi +1)}{\left(\frac{x}{2}\right)}^{2m+\xi },$$

Γ(x) is the Gamma function [35], $N_{\xi }(x)$ is the ξth-order Neumann function [35] when ξ is a nonnegative integer

(A11) $$\begin{array}{ll} N_{\xi }(x)= & -\frac{1}{\pi }{\left(\frac{x}{2}\right)}^{-\xi }\sum_{k=0}^{\xi -1}\frac{(\xi -k-1)!}{k!}{\left(\frac{x^{2}}{4}\right)}^{k}+\frac{2}{\pi }J_{\xi }(x)\ln\left(\frac{x}{2}\right)\\ \; & -\frac{1}{\pi }{\left(\frac{x}{2}\right)}^{\xi }\sum_{k=0}^{\infty }\left(\psi (k+1)+\psi (\xi +k+1)\right)\frac{1}{k!(\xi +k)!}{\left(-\frac{x^{2}}{4}\right)}^{k}, \end{array}$$

and ψ(x) is the digamma function [35].
